# Current research hotspots and difficulties of chronic prostatitis/chronic pelvic pain syndrome: neuroendocrine mechanism

**DOI:** 10.1080/07853890.2026.2616886

**Published:** 2026-01-19

**Authors:** Bo Shao, Kaixiu Wu, Zhengkai Fan, Xingwu Gao, Shui Wan, Li Xiao, Yanggen Zuo, Jinbo Pi, Pingping Sun

**Affiliations:** a Zhaotong Hospital of Traditional Chinese Medicine, Urology Department, Zhaotong City, Yunnan Province, China; b First Affiliated Medical College, Dali University, Dali City, Yunnan Province, China; c Urology Department, Wuhu First People’s Hospital, Wuhu City, Anhui Province, China

**Keywords:** Central sensitization, chronic prostatitis/chronic pelvic pain syndrome, neuroendocrine mechanisms, neuroimmune interactions, neuropeptides

## Abstract

**Background:**

Chronic prostatitis/chronic pelvic pain syndrome (CP/CPPS) presents a significant clinical challenge in urology. Traditional pathophysiological models emphasize infection and local inflammation; however, the limited efficacy of conventional therapies suggests the involvement of deeper regulatory mechanisms.

**Methods:**

This comprehensive review synthesizes evidence from the past five years regarding neuroendocrine pathways in CP/CPPS. We systematically analyzed literature from the PubMed, Web of Science, and CNKI databases, focusing on neuropeptide functions, hypothalamic–pituitary–adrenal (HPA) axis dysregulation, sympathetic nervous system (SNS) signaling, glial cell activation, and gut–prostate axis interactions.

**Results:**

Neuroendocrine mechanisms significantly contribute to the pathophysiology of CP/CPPS through multiple pathways. Substance P and calcitonin gene-related peptide promote neurogenic inflammation, while B-type natriuretic peptide exhibits analgesic effects. Dysregulation of the HPA axis and sympathetic overactivation create stress-related imbalances. Central glial cell activation leads to central sensitization, and the emerging concept of the gut–prostate axis reveals bidirectional neuroendocrine-immune communication.

**Conclusions:**

CP/CPPS is a systemic condition involving complex neuro-endocrine-immune interactions. Therapeutic strategies targeting neuroendocrine mechanisms—including neuropeptide receptor antagonists, glial cell inhibitors, and neuromodulation techniques—offer promising directions for precision medicine. Future research should focus on multi-omics approaches and neuroendocrine-based patient classification for individualized treatment.

## 1. Introduction

Chronic prostatitis is a prevalent condition within the field of urological andrology. The National Institutes of Health (NIH) classifies it into four distinct types, with type III chronic prostatitis, also known as CP/CPPS, comprising approximately 90% to 95% of all chronic prostatitis cases [1]. The hallmark symptoms of CP/CPPS include pelvic pain or discomfort persisting for over three months, frequently accompanied by various urinary symptoms (such as increased frequency, urgency, and dysuria) and sexual dysfunction (including pain during ejaculation and erectile dysfunction). These symptoms significantly impair the quality of life for patients, leading to considerable psychological and economic burdens [2].

The etiology and pathogenesis of CP/CPPS are exceedingly complex. Traditionally, these conditions are attributed to a multitude of factors, including latent infections by pathogens, immune inflammatory responses, and psychological as well as neurological disorders [3–5]. However, antibiotic therapy alone proves ineffective for the majority of patients. While anti-inflammatory medications may provide partial symptom relief, they often fail to achieve a cure and are associated with frequent relapses. This therapeutic conundrum strongly indicates the existence of a deeper regulatory mechanism that transcends classical inflammatory pathways. It is noteworthy, Recent findings from the Multidisciplinary Chronic Pelvic Pain Research Network (MAPP) have confirmed a diverse array of somatic symptoms unrelated to the urinary system, as well as alterations in the structure and function of the central nervous system [6]. Serving as a ‘bridge’ between the brain and peripheral organs, the neuroendocrine system plays a crucial role in modulating immune, inflammatory, pain, and stress responses. Recent evidence increasingly suggests that neuroendocrine disorders are pivotal in the onset and progression of CP/CPPS [7]. The nervous system interacts directly with immune cells by releasing neurotransmitters and neuropeptides, such as substance P (SP), calcitonin gene-related peptide (CGRP) and B-type natriuretic peptide (BNP), which amplify and sustain local inflammatory responses. Chronic psychological stress alters the local microenvironment by activating the HPA axis and the SNS, promoting neurogenic inflammation and tissue fibrosis [8,9]. Furthermore, the activation of glial cells in the central nervous system leads to central sensitization, which lowers the pain perception threshold in patients, resulting in persistent pain even after the initial stimulus has ceased [10]. Therefore, this article aims to comprehensively review and summarize recent research on the neuroendocrine mechanisms underlying CP/CPPS. It will analyze core issues such as the neuroimmune interactive dialogue, dysregulation of the stress system, and central sensitization. Furthermore, it will propose new treatment strategies based on these mechanisms, thereby providing novel insights and directions for further research and clinical application in this field.

## 2. The interaction between CP/CPPS and neuroimmunity

**Figure 1.**
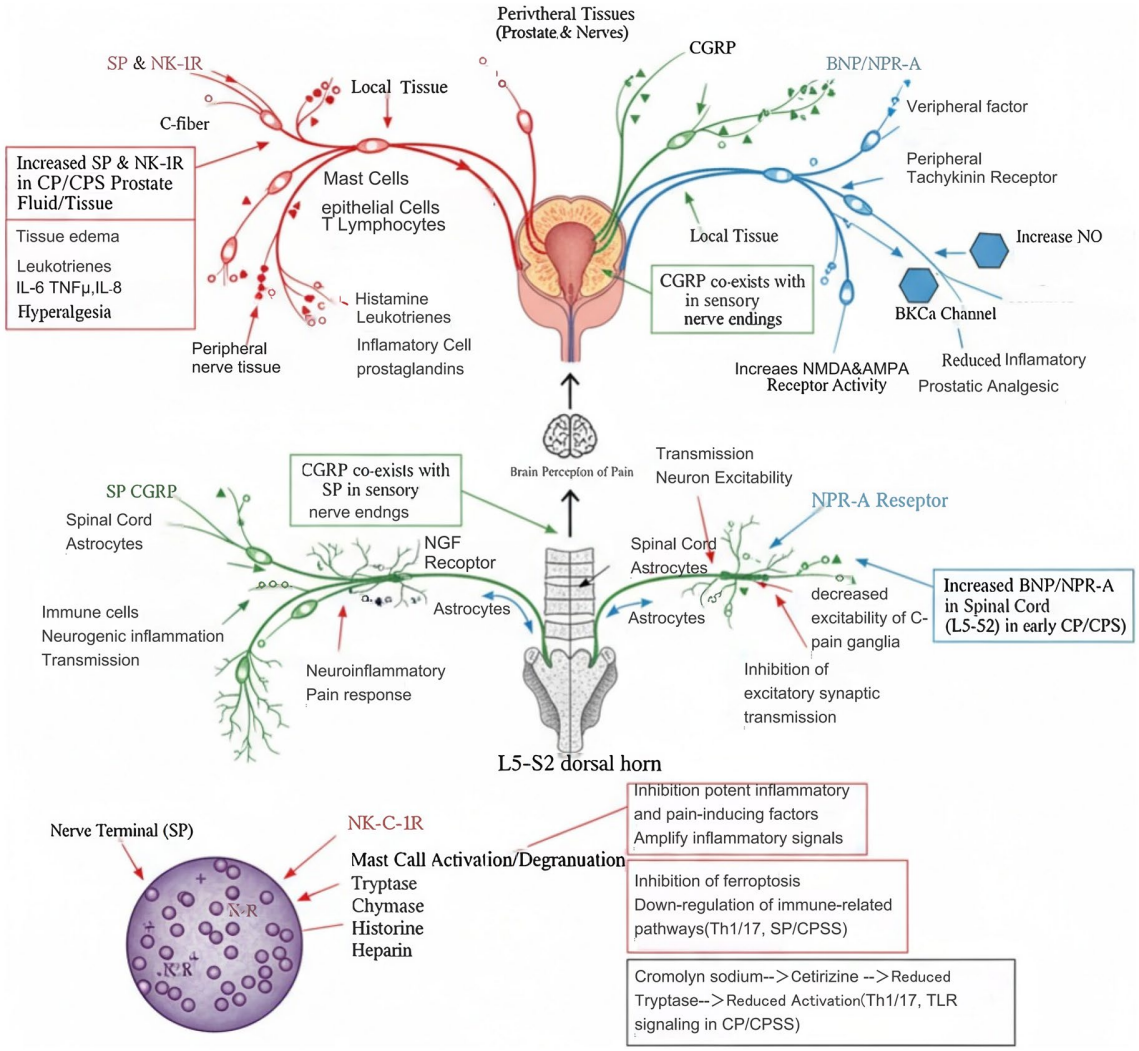
The interaction between CP / CPPS and neuroimmunity. This figure illustrates the complex neuroimmune interactions in CP/CPPS, highlighting the roles of key neuropeptides (SP, CGRP, BNP) and mast cells in mediating neurogenic inflammation. The diagram shows how sensory nerve fibers release neuropeptides that activate immune cells, leading to the release of inflammatory mediators and perpetuating the inflammatory cycle.

Neuroimmune interactions are central to the neuroendocrine mechanisms underlying CP/CPPS. Prostate tissue is characterized by a rich network of autonomic and sensory nerve fibers. These nerve endings not only receive signals from surrounding tissues but also actively modulate local immune responses and inflammatory conditions through the release of various neuropeptides. This phenomenon is referred to as ‘neurogenic inflammation.’ Understanding these neuroimmune interactions provides critical insights into the persistent pain and inflammation characteristic of CP/CPPS, as shown in Figure 1.

### 2.1. The role of key neuropeptides

#### 2.1.1. SP and NK-1R

SP is predominantly found in the C fibers of the spinal cord and serves as a critical neurotransmitter in the transmission of pain. Upon the occurrence of noxious stimulation, SP is released in both central and peripheral segments, where it binds to the NK-1R to exert its physiological effects. SP released from the central segment can directly or indirectly facilitate the transmission of pain by promoting the release of glutamate. Additionally, SP may be released simultaneously in both central and peripheral segments, interacting with NK-1R and thereby inducing a neurogenic inflammatory response. Consequently, SP is intricately linked to the development of inflammatory pain, hyperalgesia, and neuropathic pain. Clinical studies have confirmed elevated SP and NK-1R levels in prostatic fluid and biopsy tissues from CP/CPPS patients, correlating with pain severity and urinary symptoms [10]. By binding to NK-1R on immune cells, including mast cells, macrophages, T lymphocytes, and epithelial cells, SP facilitates the release of inflammatory mediators such as histamine, leukotrienes, and prostaglandins, while also upregulating the expression of cytokines such as tumor necrosis factor-alpha (TNF-α), interleukin-6 (IL-6), and interleukin-8 (IL-8). This cascade of events results in tissue edema and inflammatory cell infiltration [11]. Furthermore, SP is widely distributed throughout the central and peripheral nervous systems as well as in various tissues and organs, and it is closely associated with C-type nerve fibers in sensory nerves. Under local stimulation from CP/CPPS, C-type nerve fibers innervating the prostate and bladder can increase, store, and release SP, thereby promoting vasodilation, plasma extravasation, increasing vascular permeability, and contributing to neurogenic inflammation [12]. Animal experiments have confirmed that the induction of SP promotes the activation of spinal astrocytes, which can lead to a neuroinflammatory pain response in the L5-S2 spinal cord segment. This response contributes to inflammation and pain associated with CP/CPPS [13].

#### 2.1.2. CGRP

CGRP frequently coexists with SP in sensory nerve endings and plays a multifaceted role in CP/CPPS. CGRP is a pain-related neurotransmitter released from peptidergic nerve endings, primarily synthesized and expressed in the spinal dorsal horn. It is currently acknowledged as a neuroactive substance intricately involved in the transmission of nociceptive information and plays a significant role in pain transmission and the development of hyperalgesia [14]. Studies have demonstrated that the mechanism by which CGRP promotes nociceptive signal transduction and the development of hyperalgesia may be associated with CGRP’s role in increasing nitric oxide (NO) release and enhancing the activity of *N*-methyl-d-aspartate (NMDA) and α-amino-3-hydroxy-5-methyl-4-isoxazolepropionic acid receptors (AMPAR) [15]. The release of NO reduces the pain threshold and facilitates the onset of pain. NMDA and AMPA receptors are among the excitatory amino acid receptors located in the spinal cord. By enhancing the activity of NMDA and AMPA receptors, CGRP increases the excitability of spinal dorsal horn neurons, thereby promoting pain hypersensitivity. Additionally, nerve growth factor (NGF) serves as a crucial link between inflammation and hyperalgesia. During tissue inflammation, NGF can directly act on peripheral sensory nerve endings, binding to receptors and facilitating their transport to the spinal dorsal horn. This process upregulates the expression of CGRP and accelerates the onset of hyperalgesia. The role of NGF in prostatitis-related pain is particularly significant. Some studies indicate that increased NGF levels in prostate tissue may induce neurogenic inflammation, playing a vital role in the onset and progression of CP/CPPS pain. Therefore, CGRP may be involved in the occurrence and maintenance of CP/CPPS pain through direct or indirect ways [16].

#### 2.1.3. Analgesic effect of BNP/NPR-A on CP/CPPS

BNP is primarily synthesized by cardiomyocytes, with NPR-A serving as one of its principal receptors. Recent studies indicate that BNP predominantly exerts an analgesic effect in CP/CPPS. Studies have demonstrated that stimulation of nociceptive sensory nerve fibers induces the secretion of BNP at their terminals. This peptide interacts with the presynaptic NPR-A receptor, which inhibits excitatory synaptic transmission and reduces the excitability of spinal dorsal horn ganglion neurons, ultimately leading to pain inhibition. This phenomenon may be attributed to an increase in the levels of BNP and its receptor following bodily stimulation. The interaction between BNP and its receptor can enhance the production of cyclic guanosine monophosphate (cGMP), which subsequently activates protein kinase G1 (PKG). This activation increases the opening rate of large-conductance calcium-activated potassium channels (BKCa), resulting in decreased excitability of C-pain ganglia, thereby inhibiting excitatory synaptic transmission. Consequently, this mechanism weakens the transmission of inflammatory stimulation signals and plays a significant role in prostate analgesia [17]. Chen [18] found that intrathecal injection of a BKca activator in the spinal cord can reduce the conduction of nociceptive nerve fibers, while BNP exhibits a similar effect to the BKca activator. Xie [19] observed that the elevated expression of BNP/NPR-A is likely a result of the body’s inhibitory response to stimulation in the early stages of pain, which diminishes after later pain sensitization, leading to a corresponding decrease in BNP/NPR-A expression. Therefore, in the L5 to S2 segments of the spinal cord, BNP/NPR plays a crucial role in the analgesic mechanism related to the prostate and will be an important target for future research on the treatment of prostatitis pain.

### 2.2. Interaction between mast cells and neuropeptides

Mast cells serve as sentinel cells in neuroimmune interactions, expressing a variety of neuropeptide receptors, including NK-1R, on their surface. SP released from nerve endings can directly activate mast cells, resulting in degranulation and the release of tryptase, chymotrypsin, histamine, heparin, and other substances. These substances are potent inflammatory and pain-inducing factors that can further activate other immune cells and amplify inflammatory signals [20]. Recent studies have confirmed that the inhibition of ferroptosis can reduce the degree of mast cell activation, thereby decreasing the levels of inflammatory factors in prostate tissue [21]. Furthermore, research has demonstrated that in patients with chronic prostatitis, treatment with cromolyn sodium and cetirizine dihydrochloride leads to a decrease in the concentration of tryptase in mast cells. This reduction is associated with the down-regulation of immune-related pathways in the microenvironment, including Th1 and Th17 T cell differentiation and TLR signaling [22]. These findings suggest that the abnormal activation of mast cells is closely linked to the onset and progression of CP/CPPS.

## 3. Stress system: HPA axis and SNS disorders

**Figure 2.**
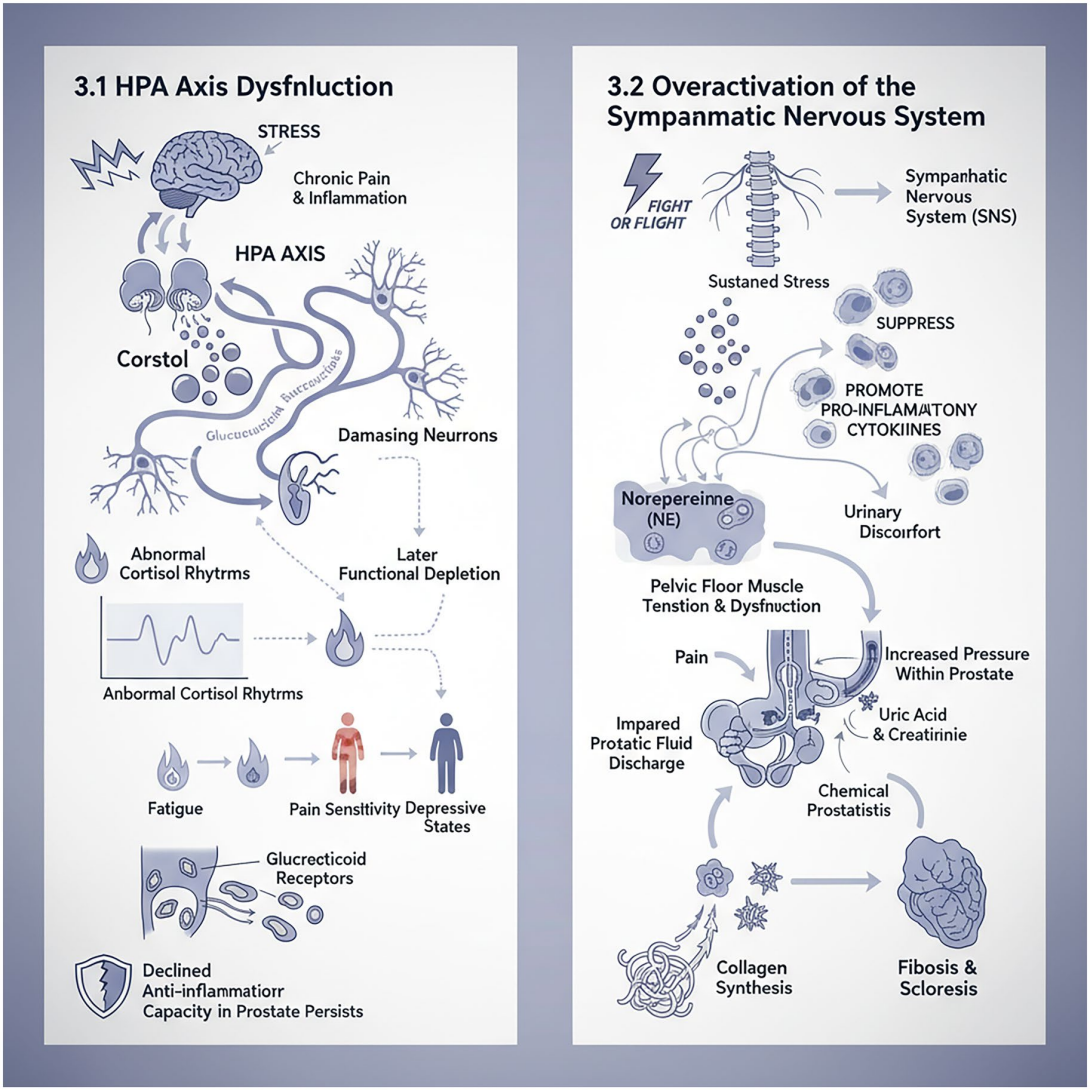
HPA axis dysfunction and overactivation of the sympathetic nervous system. This figure depicts the dysregulation of the HPA axis and SNS in CP/CPPS, showing how chronic stress leads to abnormal cortisol rhythms and sustained norepinephrine release, contributing to inflammation, fibrosis, and pelvic floor dysfunction.

Chronic stress and emotional disorders, such as anxiety and depression, are not only consequences of CP/CPPS, but also significant inducing and aggravating factors. The underlying biological basis for these conditions is the dysfunction of the HPA axis and the SNS. The dysregulation of these stress response systems contributes significantly to the pathophysiology of CP/CPPS, affecting both peripheral inflammation and central pain processing, as shown in Figure 2.

### 3.1. HPA axis dysfunction

The HPA axis is the primary neuroendocrine system responsible for the body’s response to stress. Chronic pain, stress, and inflammation can lead to hyperactivity of the HPA axis, resulting in an increased release of glucocorticoids. This hyperactivity can affect the release of downstream neurotransmitters and cytokines, damage neurons, and cause abnormal alterations in the central nervous system. These alterations are characterized by an initial phase of overactivation followed by a later phase of functional exhaustion. Research has indicated that some patients with CP/CPPS exhibit abnormal rhythms and baseline levels of cortisol, the terminal effector hormone of the HPA axis. These abnormalities are closely associated with the patients’ fatigue, pain sensitivity, and depression [23]. Dimitrakov [24] evaluated the serum levels of 12 steroids in 27 patients with CP/CPPS and found significant alterations in the adrenocortical hormones of these patients. Specifically, the levels of corticosterone, aldosterone, and 11-deoxycorticosteroid were markedly increased, while aldosterone and cortisol levels showed a correlation with both the total score and pain score on the National Institutes of Health Chronic Prostatitis Symptom Index (NIH-CPSI). Similarly, Anderson [25] measured free cortisol levels in the morning saliva of CP/CPPS patients and observed that the salivary cortisol levels after early morning awakening were significantly higher than those in the control group, indicating potential dysfunction of the HPA axis in these patients. Compared to healthy individuals, patients with CP/CPPS exhibit more pronounced psychological disorders. Stress can significantly impact the secretion of adrenocorticotropic hormone (ACTH) in these patients. The increase in serum ACTH levels before and after stress is approximately 30% lower than that observed in healthy subjects, which is reflected in a blunted response curve [26]. Šutulović [27] found that in a study involving CP/CPPS animal models, corticosterone levels were positively correlated with anxiety-like behaviors, indicating that mental disorders are influenced by adrenocortical hormones. Furthermore, glucocorticoids possess potent anti-inflammatory properties, and impaired HPA axis function results in a diminished endogenous anti-inflammatory capacity, thereby prolonging inflammation. Additionally, abnormal expression and function of glucocorticoid receptors in prostate tissue cells may contribute to their insensitivity to the anti-inflammatory effects of glucocorticoids [28].

### 3.2. Excessive activation of SNS

The ‘fight or flight’ response of the SNS serves to protect the body during acute stress; however, chronic activation can be detrimental. In cases of CP/CPPS, prolonged stress results in heightened SNS tension and continuous release of norepinephrine (NE). NE has complex immunomodulatory effects by interacting with adrenergic receptors, primarily the β2 receptors, on immune cells. On one hand, it may inhibit certain innate immune responses; on the other hand, it can enhance Th1 cell responses and the production of pro-inflammatory cytokines [29,30]. More importantly, Sustained activation of the SNS can lead to pelvic floor muscle contraction tension and dysfunction, as well as impairment of the prostatic duct and urethral sphincter. These factors can contribute to disorders in prostatic fluid excretion and urine reflux, resulting in increased pressure within the prostate. Consequently, substances such as uric acid and creatinine may reflux into the prostatic urethra, potentially inducing chemical prostatitis [31]. Furthermore, NE can directly stimulate fibroblast proliferation and collagen synthesis, thereby contributing to the fibrosis and sclerosis of prostate tissue. This phenomenon aligns with the pathological changes observed in patients with CP/CPPS, characterized by prostate hardening and reduction in volume [32].

## 4. Central sensitization and glial cell activation

**Figure 3.**
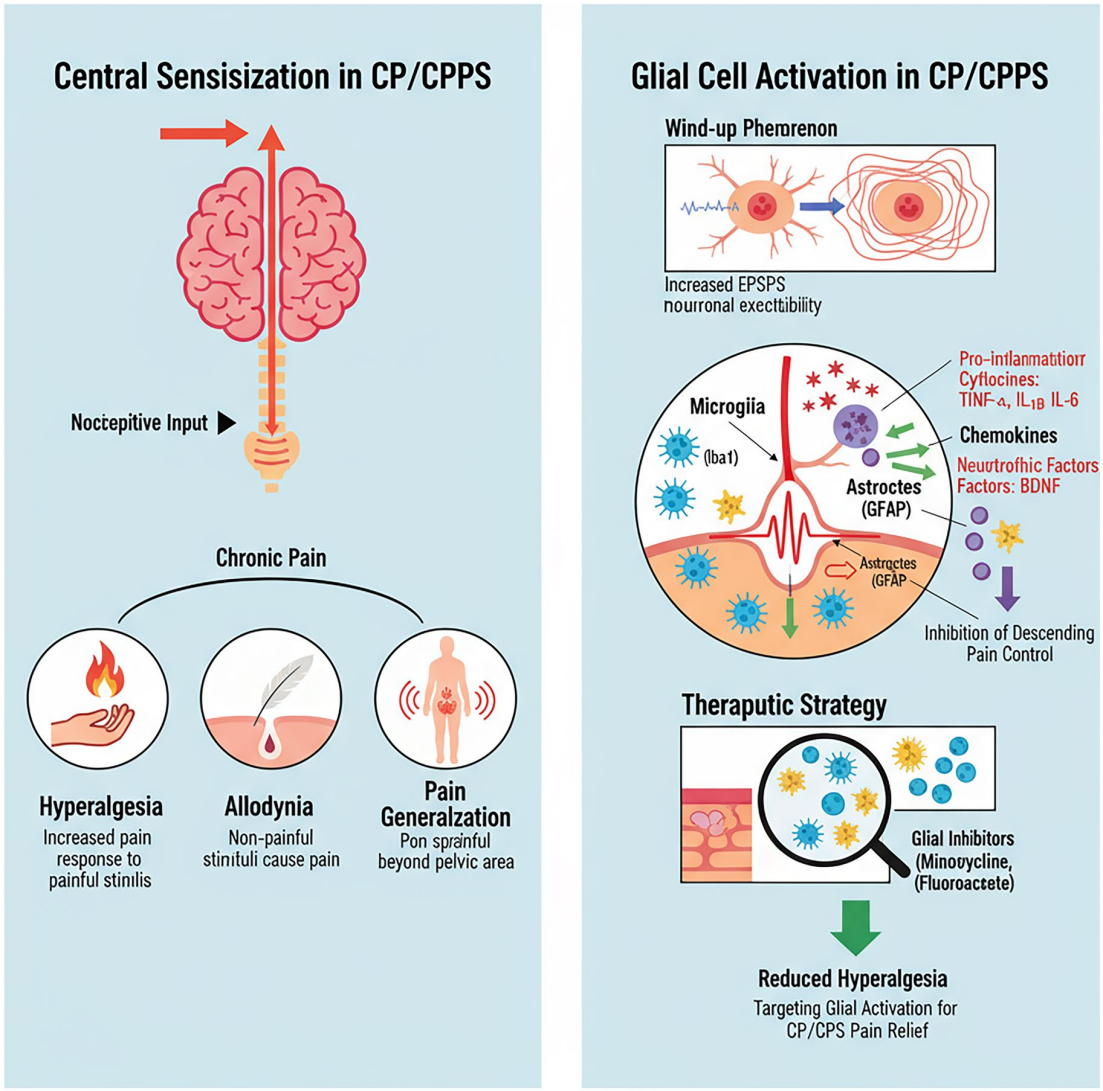
Central sensitization and glial cell activation. This figure illustrates the mechanisms of central sensitization in CP/CPPS, showing how persistent nociceptive input leads to glial cell activation in the spinal cord, resulting in the release of pro-inflammatory cytokines and neurotrophic factors that maintain chronic pain states.

The nature of chronic pain in CP/CPPS extends far beyond mere peripheral tissue damage, with the plasticity of the central nervous system (CNS) playing a decisive role in its manifestation. Central nervous system changes, including central sensitization and glial cell activation, are now recognized as key contributors to the chronic pain experienced by CP/CPPS patients, as shown in Figure 3.

### 4.1. Central sensitization

Central sensitization refers to the persistent enhancement of spinal cord and cerebral neuron reactivity to afferent pain signals. Prolonged peripheral inflammation and nociceptive signal input lead to the ‘wind-up’ phenomenon in spinal dorsal horn neurons, characterized by the continuous superposition of excitatory postsynaptic potentials. This phenomenon ultimately lowers the response threshold of neurons to subsequent stimuli, increases response intensity, and expands the receptive field [33]. Consequently, patients with CP/CPPS exhibit the following symptoms: 1) Hyperalgesia: an exaggerated response to painful stimuli; 2) Allodynia: non-painful stimuli (such as light touch or dressing) can also elicit pain; and 3) Pain generalization: pain may extend beyond the pelvic cavity, affecting areas such as the abdomen and thighs.

### 4.2. Glial cell activation

Historically, pain treatment was thought to be solely associated with neuronal activity. However, contemporary research indicates that the activation of central glial cells—particularly microglia and astrocytes—plays a crucial role in promoting central sensitization. When activated by peripheral inflammatory signals, these glial cells release a significant quantity of pro-inflammatory cytokines (such as TNF-α, IL-1β, and IL-6), chemokines, and neurotrophic factors (including BDNF). Collectively, these substances enhance neuronal excitability and inhibit the descending pain inhibition pathways, thereby sustaining and amplifying pain signals [34]. In the study of CP/CPPS animal models, it was observed that the expression of microglial and astrocyte markers (Iba1, GFAP) in the lumbosacral segment of the spinal cord was significantly upregulated. Furthermore, the application of glial cell inhibitors, such as minocycline and fluorocitrate, effectively reduced mechanical hyperalgesia [35]. This finding supports the notion that targeted inhibition of central glial cell activation represents a promising new strategy for the treatment of CP/CPPS pain.

## 5. New frontier: neuroendocrine connection of gut–prostate axis

**Figure 4.**
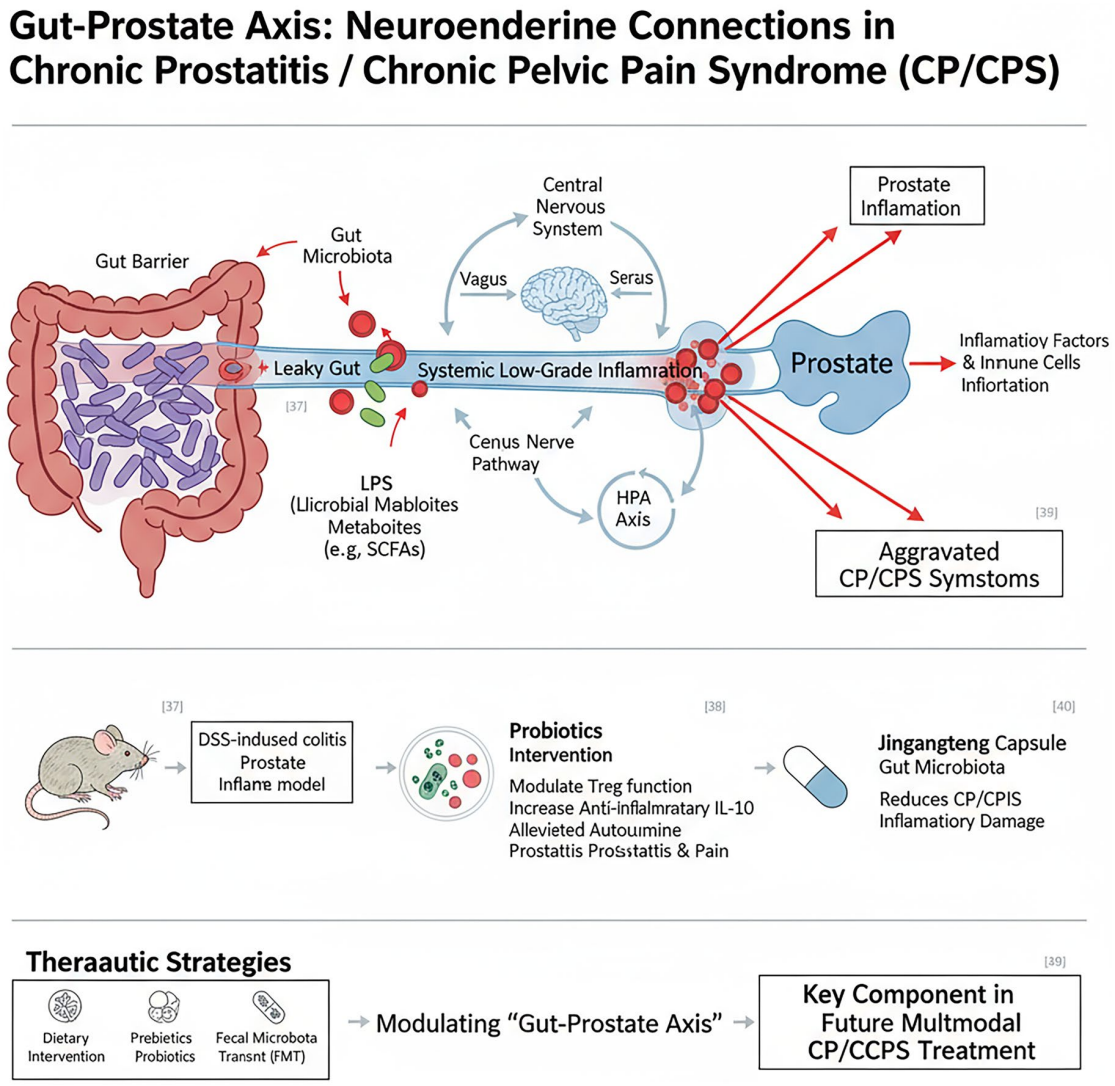
Neuroendocrine connection of gut–prostate axis. This figure illustrates the proposed gut–prostate axis in CP/CPPS, demonstrating how gut dysbiosis and impaired intestinal barrier function can lead to systemic inflammation that affects prostate health, while also showing the bidirectional communication between the gut microbiota and the central nervous system.

Recent studies have underscored the pivotal regulatory role of gut microbiota in systemic immunity and the functioning of the nervous system. Building upon the concept of the ‘gut–brain axis’, we propose the hypothesis of a ‘gut–prostate axis’, offering a novel perspective for understanding CP/CPPS. This emerging concept suggests bidirectional communication between the gut microbiome and prostate health through neuroendocrine pathways, as shown in Figure 4.

An imbalance in intestinal flora may compromise the integrity of the intestinal barrier, leading to a phenomenon commonly referred to as ‘intestinal leakage’. This condition allows microbial-related molecules, such as bacterial lipopolysaccharides (LPS), to enter the bloodstream, thereby triggering a systemic low-grade inflammatory response [36]. These inflammatory mediators and immune cells can infiltrate the prostate, exacerbating local inflammation. Importantly, the intestinal flora interacts with the central nervous system through metabolites, such as short-chain fatty acids (SCFAs), and via vagus nerve pathways, influencing the HPA axis stress response and neuroimmune function [37]. Mice with colitis induced by experimental methods often exhibit complications such as prostatic inflammation. However, probiotic interventions have been shown to significantly alleviate both the degree of inflammation and pain behaviors associated with experimental autoimmune prostatitis by modulating the function of regulatory T cells (Treg) and increasing the levels of the anti-inflammatory factor IL-10 [38]. Furthermore, research indicates that Jingangteng capsules can mitigate inflammatory damage in CP/CPPS by regulating intestinal flora [39]. The latest findings from the MAPP study confirm a broad spectrum of somatic symptoms unrelated to the urinary system, as well as alterations in the structure and function of the central nervous system. These results lend support to the gut–prostate axis hypothesis and imply that interventions aimed at the neuroimmune mechanisms of this system may be especially advantageous [6]. This suggests that the regulation of the ‘gut–prostate axis’ through dietary modifications, prebiotics/probiotics, or fecal microbial transplantation may play a crucial role in the multimodal treatment of CP/CPPS in the future.

## 6. Prospect of therapeutic strategies targeting neuroendocrine mechanisms

The in-depth understanding of the neuroendocrine mechanisms underlying CP/CPPS has led to the development of several potential new treatment strategies. These strategies include neuropeptide receptor antagonists, modulators of the HPA axis and SNS, glial cell inhibitors, neuromodulation therapies, as well as lifestyle modifications and psychological interventions.

Neuropeptide receptor antagonists, such as NK-1R antagonists (e.g. aprepitant), can effectively block the pathogenic pathway of SP and inhibit neurogenic inflammation at its source. Preliminary clinical studies have demonstrated that these antagonists can significantly reduce pain and inflammatory responses, indicating a promising direction for therapeutic transformation [40]. Alpha-adrenergic receptor blockers, such as tamsulosin, are currently considered first-line treatment options. Their mechanism of action involves not only the relaxation of the bladder neck and prostate smooth muscle to enhance urination but also the inhibition of immune responses and fibrosis by blocking SNS overactivation [41]. Cortisol modulators and drug therapies for stress-related psychiatric comorbidities, such as selective serotonin reuptake inhibitors (SSRIs) and serotonin-norepinephrine reuptake inhibitors (SNRIs) like duloxetine, not only improve mood but also exhibit central analgesic effects and regulate neurotransmitters. These mechanisms are essential for breaking the ‘pain–stress’ vicious cycle [42]. Minocycline has demonstrated pain relief by inhibiting microglial activation in animal models, while other drugs, such as propentofylline, are currently under development. For patients with severe conditions and inadequate response to pharmacological treatments, neuromodulation techniques, including peripheral tibial nerve stimulation (PTNS) and sacral nerve modulation (SNM), offer promising alternatives. The proposed mechanism involves rebalancing pelvic floor nerve reflexes by modulating the afferent and efferent signals of the sacral nerve, thereby alleviating pain and lower urinary tract symptoms [7]. Psychotherapy techniques, including cognitive behavioral therapy (CBT) and mindfulness-based stress reduction (MBSR), have been shown to effectively reduce patients’ stress levels. This reduction can indirectly regulate the HPA axis and SNS function, thereby alleviating associated symptoms [43]. Furthermore, regular exercise and a healthy diet—such as a high-fiber diet to enhance intestinal flora—serve as fundamental components in a multimodal management approach [44].

Furthermore, from a clinical perspective, the UPOINTS (Urinary, Psychosocial, Organ-specific, Infection, Neurological/Systemic, Tenderness of Skeletal Muscle) classification system provides a useful framework for phenotyping CP/CPPS patients and guiding targeted therapy. Neuroendocrine mechanisms are particularly relevant to the Psychosocial, Neurological/Systemic, and Tenderness domains of UPOINTS. Patients with prominent neuroendocrine dysregulation may benefit from a tailored approach that addresses their specific mechanistic profile, such as NK-1R antagonists for those with elevated SP levels, glial cell modulators for those with central sensitization features, or HPA axis regulators for those with stress-related symptoms. This phenotyping approach represents a shift toward precision medicine in CP/CPPS management [45].

## 7. Summary and outlook

In summary, CP/CPPS is not merely a localized prostate organ disease; rather, it represents a systemic condition that involves the intricate interplay among the nervous, endocrine, and immune systems. The neuroendocrine mechanisms are integral throughout the entirety of its pathophysiology. This process begins with the release of neuropeptides from peripheral sensory nerves, leading to neurogenic inflammation. Subsequently, there is an imbalance in SNS and the HPA axis, which contributes to the persistence of inflammation and fibrosis. Furthermore, the activation of central glial cells results in central sensitization and chronic pain. The emerging concept of the ‘gut–prostate axis’ adds another dimension to this discussion, reinforcing the notion that neuroendocrine mechanisms are central to the entire process, with each component forming a complex network system.

Future studies should prioritize the application of multi-omics techniques—such as transcriptomics, proteomics, and metabolomics—to more accurately delineate the neuroendocrine molecular landscape of CP/CPPS while identifying specific biomarkers. Concurrently, there is a need for the development of more targeted therapeutics, including highly effective NK-1R antagonists with minimal side effects and specific glial cell modulators. Furthermore, it is essential to investigate the mechanisms underlying the ‘gut–prostate axis’, assess the feasibility of microbiome therapy, and formulate precise treatment strategies for CP/CPPS based on neuroendocrine classification to facilitate ‘individualized treatment.’ A profound understanding of the neuroendocrine mechanisms underlying CP/CPPS will facilitate a paradigm shift in treatment approaches, moving away from traditional strategies focused on ‘anti-infection and anti-inflammation’ towards ‘regulation of neuroimmunity and restoration of systemic balance.’ This shift ultimately holds the promise of renewed hope for the numerous patients who suffer from this condition.

## Data Availability

Data sharing is not applicable to this article as no new data were created or analyzed in this study.
